# ELK1 is up-regulated by androgen in bladder cancer cells and promotes tumor progression

**DOI:** 10.18632/oncotarget.5007

**Published:** 2015-08-22

**Authors:** Takashi Kawahara, Hasanain Khaleel Shareef, Ali Kadhim Aljarah, Hiroki Ide, Yi Li, Eiji Kashiwagi, George J. Netto, Yichun Zheng, Hiroshi Miyamoto

**Affiliations:** ^1^ Departments of Pathology and Urology, Johns Hopkins University School of Medicine, Baltimore, MD, USA; ^2^ Department of Pathology and Laboratory Medicine, University of Rochester Medical Center, Rochester, NY, USA; ^3^ Department of Urology, Yokohama City University School of Medicine, Yokohama, Japan; ^4^ Department of Biology, University of Babylon College of Science for Women, Babylon, Iraq; ^5^ Department of Biology, University of Baghdad College of Science, Baghdad, Iraq; ^6^ Department of Urology, 2nd Affiliated Hospital of Zhejiang University School of Medicine, Hangzhou, China

**Keywords:** androgen, bladder cancer, ELK1, immunohistochemistry, tumor progression

## Abstract

Little is known about biological significance of ELK1, a transcriptional factor that activates downstream targets including *c-fos* proto-oncogene, in bladder cancer. Recent preclinical evidence also suggests the involvement of androgen receptor (AR) signaling in bladder cancer progression. In this study, we aim to investigate the functions of ELK1 in bladder cancer growth and their regulation by AR signals. Immunohistochemistry in bladder tumor specimens showed that the levels of phospho-ELK1 (p-ELK1) expression were significantly elevated in urothelial neoplasms, compared with non-neoplastic urothelium tissues, and were also correlated with AR positivity. Patients with p-ELK1-positive non-muscle-invasive and muscle-invasive tumors had significantly higher risks for tumor recurrence and progression, respectively. In AR-positive bladder cancer cell lines, dihydrotestosterone treatment increased ELK1 expression (mRNA, protein) and its nuclear translocation, ELK1 transcriptional activity, and c-fos expression, which was restored by an anti-androgen hydroxyflutamide. ELK1 silencing via short hairpin RNA (shRNA) resulted in decreases in cell viability/colony formation, and cell migration/invasion as well as an increase in apoptosis. Importantly, ELK1 appears to require activated AR to regulate bladder cancer cell proliferation, but not cell migration. Androgen also failed to significantly induce AR transactivation in ELK1-knockdown cells. In accordance with our *in vitro* findings, ELK1-shRNA expression considerably retarded tumor formation as well as its growth in xenograft-bearing male mice. Our results suggest that ELK1 plays an important role in bladder tumorigenesis and cancer progression, which is further induced by AR activation. Accordingly, ELK1 inhibition, together with AR inactivation, has the potential of being a therapeutic approach for bladder cancer.

## INTRODUCTION

Increasing preclinical evidence suggests a critical role of steroid hormone receptor signals in the development and progression of urothelial carcinoma [1]. In particular, we and others have demonstrated the data indicating the promotion of bladder cancer growth by androgen-mediated androgen receptor (AR) activation [2–11]. Androgens have also been shown to modulate the expression or activity of some molecules related to cell proliferation and/or tumor growth, such as β-catenin, CD24, epidermal growth factor receptor, and extracellular signal-regulated kinases (ERK), via the AR pathway. These available data thus support that targeting androgens or AR provides effective therapeutic approaches for advanced bladder cancer. However, the underlying mechanisms of how AR signals regulate bladder cancer growth remain far from fully understood.

As a transcription factor, ETS domain-containing protein ELK1 regulates the expression of a variety of genes, including a proto-oncogene *c-fos* [12]. ELK1 is phosphorylated through activation of the MAPK/ERK pathways and translocates to the nucleus, resulting in activation of downstream targets [13, 14]. Of note is that ELK1 regulates the activity of genes associated with the actin cytoskeleton [15]. ELK1 has also been shown to regulate the expression of molecules engaged in the proteolysis of extracellular matrix, such as matrix metalloproteinase (MMP)-2 and MMP-9 [16]. Consequently, ELK1 is able to control cell migration and invasion [15–17]. The involvement of ELK1 signals in cancer development, possibly via the regulation of inflammatory responses, has also been documented [18].

Recently, in prostate cancer cells where the role of AR signals had been extensively studied, AR was found to function as a coactivator of ELK1 [19]. Indeed, significant growth retardation was seen in androgen-sensitive, AR-positive prostate cancer LNCaP cells expressing ELK1-short hairpin RNA (shRNA), compared with control cells, cultured in the presence of androgen [19]. In the current study, we investigated whether androgen could activate ELK1, as a downstream target of AR, in bladder cancer cells as well as whether ELK1 could affect their proliferation and migration in the presence and absence of androgen.

## RESULTS

### Transcription factors up-regulated by androgen in bladder cancer cells

We aimed to identify downstream targets of androgen-mediated AR signaling in bladder cancer cells. Using a profiling array kit, activities of 96 known transcription factors were compared in AR-positive bladder cancer UMUC3 cells with versus without a non-aromatizable synthetic androgen methyltrienolone (R1881) treatment. Of the 96 transcription factors, six were found to be induced (*i.e*. greater than 3.0-fold increase) by the androgen. These included nuclear factor of activated T-cells (NFAT; 6.3-fold), NKX2–5 (4.3-fold), ELK1 (4.1-fold), RAR-related orphan receptor (ROR; 3.5-fold), GLI1 (3.3-fold), and MyoD (3.2-fold). Additionally, in this assay, R1881 up-regulated AR by 1.7-fold.

**Figure 1:**
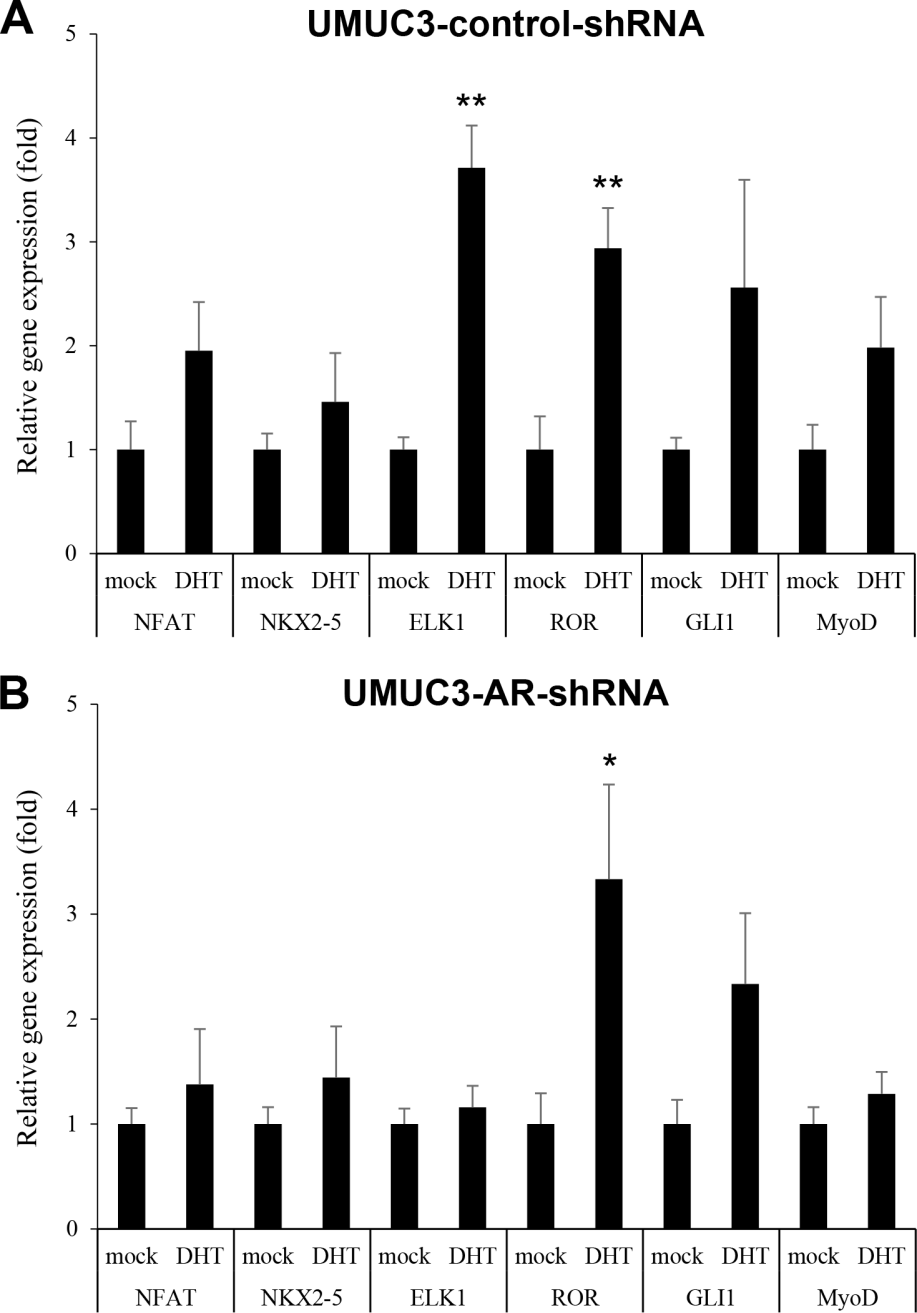
Effects of androgen on the expression of transcriptional factors in bladder cancer cells UMUC3-control-shRNA **A.** or UMUC3-AR-shRNA **B.** treated with ethanol (mock) or 1 nM DHT for 24 hours were subjected to RNA extraction and subsequent real-time RT-PCR of *NFAT*, *NKX2–5*, *ELK1*, *ROR*, *GLI1*, and *MyoD*. Expression of each gene was normalized to that of *GAPDH*. Transcription amount is presented relative to that of mock treatment in each cell line. Each value represents the mean (+SD) from at least three independent experiments. *P < 0.05 (*vs*. mock treatment). **P < 0.01 (*vs*. mock treatment).

Changes in the expression levels of these six genes up-regulated in the profiling assay were next examined in UMUC3-control-shRNA (Figure 1A) and UMUC3-AR-shRNA (Figure 1B) treated with 1 nM dihydrotestosterone (DHT) for 24 hours. Androgen treatment significantly increased the expression of two of the transcription factors, ELK1 (3.7-fold) and ROR (2.9-fold), in control cells, while others were marginally up-regulated (1.6–2.5-fold, *P* > 0.05). In AR knockdown cells, DHT still significantly induced ROR expression (3.3-fold), whereas it only marginally increased ELK1 expression (1.2-fold). These results suggested that androgens could up-regulate ELK1 expression through the AR pathway in bladder cancer cells. We therefore decided to further study ELK1 as a potential target of androgen/AR signals in bladder cancer.

### Expression of ELK1 in human bladder cancer

**Figure 2:**
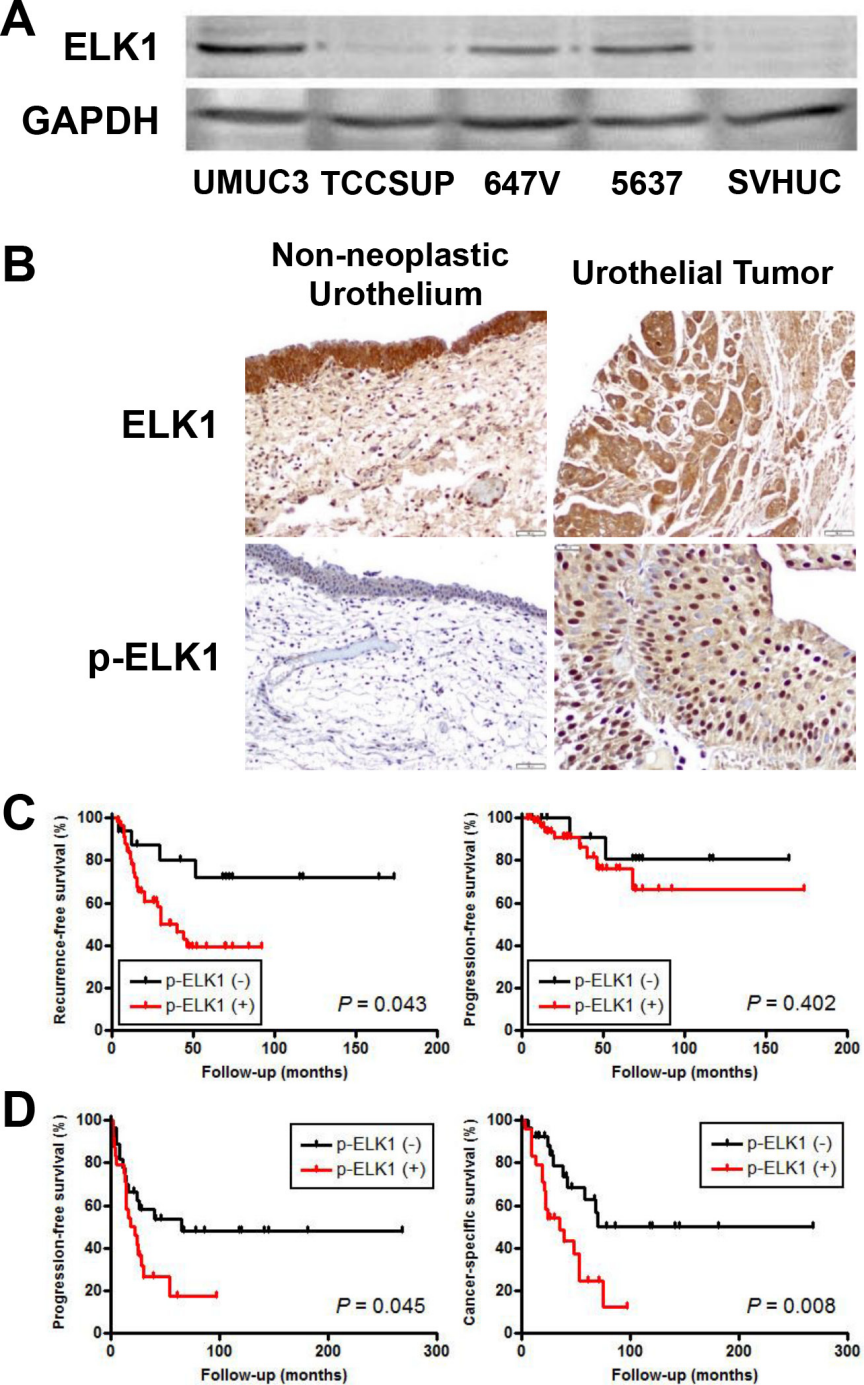
ELK1 expression in bladder cancer **A.** Western blotting of ELK1 in urothelial cell lines. Total protein extracted from each cell line was immunoblotted for ELK1 (62 kDa). GAPDH (37 kDa) served as an internal control. **B.** IHC of ELK1 and p-ELK1 in non-neoplastic urothelium and urothelial tumor specimens. Kaplan-Meier analyses for recurrence-free/progression-free survival in patients with non-muscle-invasive tumor **C.** as well as progression-free/cancer-specific survival in patients with muscle-invasive tumor **D.**, according to the levels of p-ELK1 expression.

We investigated the expression of ELK1 in human urothelial carcinoma cell lines, UMUC3, TCCSUP, 647V, and 5637, as well as an immortalized human normal urothelial cell line, SVHUC, by western blotting (Figure 2A). ELK1 expression was found to be the strongest in UMUC3 and the weakest in SVHUC. No significant difference in ELK1 expression between UMUC3- control-shRNA and UMUC3-AR-shRNA or between 647V-AR and 647V-control was seen (data not shown).

We also stained immunohistochemically for ELK1 and phospho-ELK1 (p-ELK1) in 129 bladder urothelial neoplasm specimens and corresponding 86 non-neoplastic bladder tissues. Positive signals of ELK1 and p-ELK1 were detected predominantly in the cytoplasm and nucleus, respectively, of benign/malignant urothelial cells (Figure 2B). Overall, ELK1/p-ELK1 was positive in 100% (24.8% 2+, 75.2% 3+)/65.9% (35.7% 1+, 14.7% 2+, 15.5% 3+) of tumors, which was significantly higher than in benign urothelial tissues [100% (8.1% 1+, 37.2% 2+, 54.7% 3+)/34.9% (20.9% 1+, 11.6% 2+, 2.3% 3+)] (Table 1). In tumors, the expression levels of ELK1 versus p-ELK1 were correlated (r^2^ = 0.465, *P* < 0.001). In addition, correlations between expression of ELK1 versus AR (r^2^ = 0.303, *P* < 0.001) as well as that of p-ELK1 versus AR (r^2^ = 0.223, *P* = 0.011) were significant. Kaplan-Meier and log-rank tests revealed that patients with p-ELK1-positive non-muscle-invasive (Figure 2C) and muscle-invasive (Figure 2D) tumors had significantly higher risks for tumor recurrence (*P* = 0.043) and disease progression (*P* = 0.045)/cancer-specific mortality (*P* = 0.008), respectively. In contrast, no significant associations between ELK1 levels in tumors and patient outcomes were found (data not shown). To determine whether p-ELK1 expression was an independent prognosticator, multivariate analysis was performed with Cox model (Table 2). In non-muscle-invasive tumors, p-ELK1 positivity and tumor recurrence showed a trend toward significance [hazard ratio (HR) = 2.829, *P* = 0.056]. In muscle-invasive tumors, p-ELK1 positivity strongly correlated with cancer-specific survival (H*R* = 2.693, *P* = 0.021), whereas positivity of AR (H*R* = 2.280, *P* = 0.042), but not that of p-ELK1, was identified as a strong predictor for disease progression. These findings in our immunohistochemistry (IHC) suggest that ELK1 activation may involve bladder tumorigenesis and cancer progression.

**Table 1:** Expression of ELK1 and p-ELK1 in bladder tissue microarrays

	*n*	Expression levels				*P*-value		
		Negative	Positive					
		0	1+	2+	3+	0 *vs* 1+/2+/3+	0/1+ *vs* 2+/3+	0/1+/2+ *vs* 3+
**ELK1**								
Non-neoplastic urothelium	86	0 (0%)	7 (8.1%)	32 (37.2%)	47 (54.7%)	NA	0.001	< 0.001
Urothelial neoplasm	129	0 (0%)	0 (0%)	32 (24.8%)	97 (75.2%)			
**p-ELK1**								
Non-neoplastic urothelium	86	56 (65.1%)	18 (20.9%)	10 (11.6%)	2 (2.3%)	< 0.001	0.008	0.002
Urothelial neoplasm	129	44 (34.1%)	46 (35.7%)	19 (14.7%)	20 (15.5%)			

Table 2Univariate and multivariate Cox regression analyses of histopathological and immunohistochemical factors

**Table:** 

Non-muscle-invasive tumors							
	Dichotomized variables	Recurrence-free survival			Progression-free survival		
		Univariate	Multivariate^a^		Univariate	Multivariate^a^	
		*P*-value	HR (95%CI)	*P*-value	*P*-value	HR (95%CI)	*P*-value
Tumor grade	LMP+LG vs HG	0.016	2.388 (1.174 – 4.856)	0.016	0.020	9.418 (1.995 – 44.457)	0.005
Pathologic T stage	pTa vs pT1	0.823			0.150		
LN involvement	NA^b^	NA			NA		
LVI	NA^c^	NA			NA		
Concomitant CIS	NA^d^	NA			NA		
AR	(−) vs (+)	0.669			0.124		
ELK1	(2+) vs (3+)	0.450			0.950		
p-ELK1	(−) vs (+)	0.043	2.829 (0.976 – 8.204)	0.056	0.402

**Table:** 

Muscle-invasive tumors							
	Dichotomized variables	Progression-free survival			Cancer-specific survival		
		Univariate	Multivariate^a^		Univariate	Multivariate^a^	
		*P*-value	HR (95%CI)	*P*-value	*P*-value	HR (95%CI)	*P*-value
Tumor grade	NA^e^	NA			NA		
Pathologic T stage	pT2 vs pT3–4	< 0.001	4.065 (1.536 – 10.757)	0.005	0.005	3.859 (1.314 – 11.337)	0.014
LN involvement	pN0 vs pN+	0.131			0.061		
LVI	(−) vs (+)	0.257			0.338		
Concomitant CIS	(−) vs (+)	0.502			0.437		
AR	(−) vs (+)	0.013	2.280 (1.032 – 5.036)	0.042	0.137		
ELK1	(2+) vs (3+)	0.170			0.538		
p-ELK1	(−) vs (+)	0.045			0.008	2.693 (1.164 – 6.229)	0.021

LN: lymph node; LVI: lymphovascular invasion; CIS: carcinoma in situ; AR: androgen receptor; LMP: papillary urothelial neoplasm of low malignant potential; LG: low-grade urothelial carcinoma; HG: high-grade urothelial carcinoma; HR: hazard ratio; CI: confidence interval

a Data for each parameter with a *P*-value of > 0.1 is not shown.

b No patients underwent LN dissection.

c No tumors exhibited LVI.

d No tumors exhibited concomitant CIS.

e All cases were high-grade carcinomas.

### Effects of androgen on ELK1 activity in bladder cancer cells

**Figure 3:**
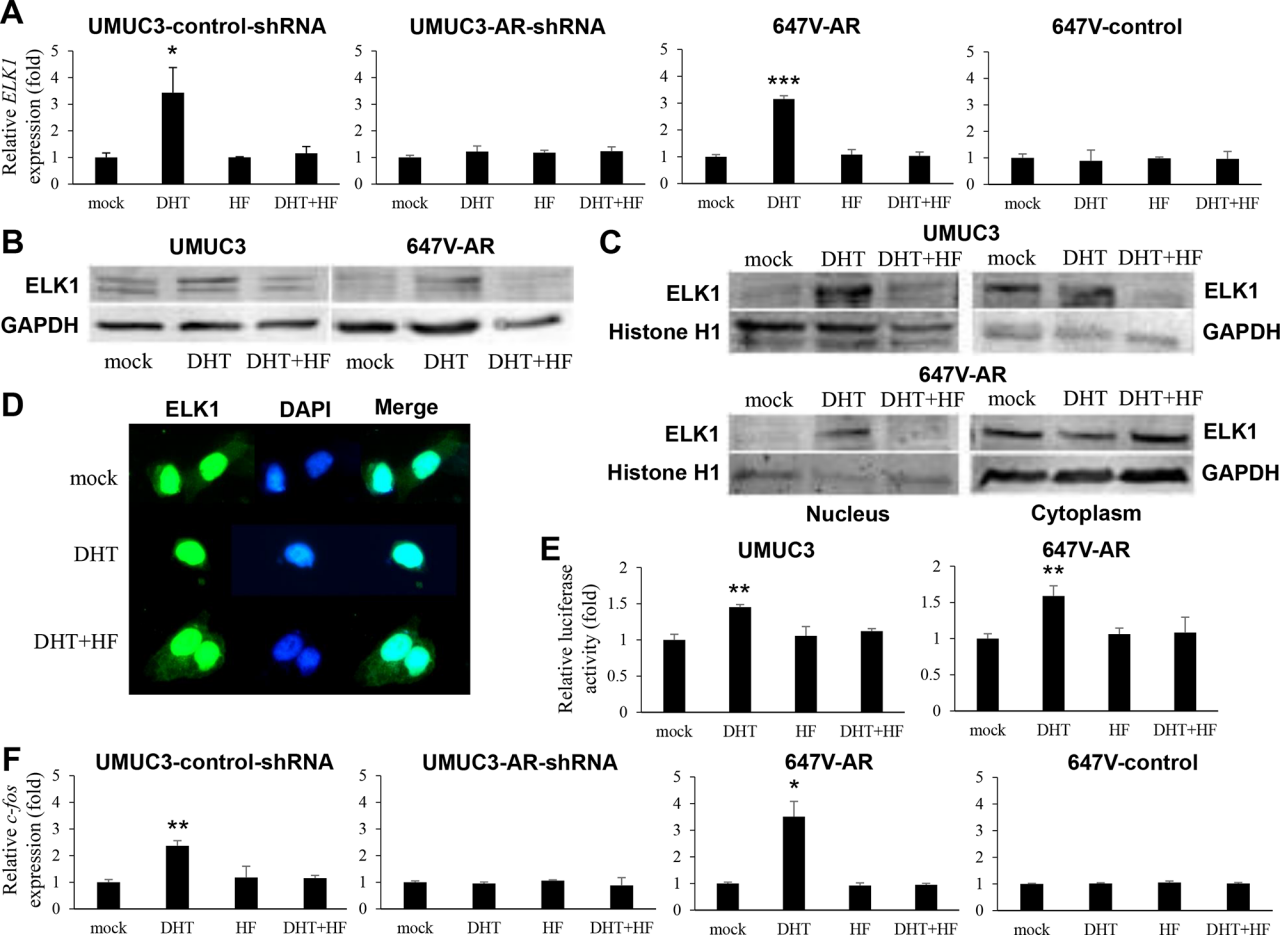
Effects of androgen on ELK1 activity in bladder cancer cells **A.** Quantitative RT-PCR of *ELK1*. UMUC3-control-shRNA/AR-shRNA and 647V-AR/control treated with ethanol (mock), 1 nM DHT, and/or 5 μM HF for 24 hours were subjected to RNA extraction and subsequent real-time RT-PCR. Expression of *ELK1* gene was normalized to that of *GAPDH*. Transcription amount is presented relative to that of mock treatment in each cell line. Each value represents the mean (+SD) from at least three independent experiments. Western blotting of ELK1 in UMUC3 and 647V-AR treated with ethanol (mock), 1 nM DHT, and/or 5 μM HF for 24 hours. Total protein extracted from each cell line **B.** or separate nuclear and cytoplasmic protein fractions **C.** were immunoblotted for ELK1 (62 kDa). Histone H1 (32–33 kDa) and GAPDH (37 kDa) served as internal controls of nuclear and cytoplasmic proteins, respectively. **D.** Immunofluorescent staining of ELK1 in UMUC3 treated with ethanol (mock), 1 nM DHT, and/or 5 μM HF for 24 hours. We merged the images between ELK1 and DAPI that was used to visualize nuclei. Cytoplasmic signals of ELK1 are seen in mock- or DHT+HF-treated cells, but not in DHT-treated cell. **E.** ELK1 luciferase reporter activity in UMUC3 and 647V-AR transfected with pELK1-Luc and pRL-TK and subsequently cultured with ethanol (mock), 1 nM DHT, and/or 5 μM HF for 24 hours. Luciferase activity is presented relative to that of mock treatment in each cell line. Each value represents the mean (+SD) from at least three independent experiments. **F.** Quantitative RT-PCR of *c-fos*. UMUC3-control/AR-shRNA and 647V-AR/control treated with ethanol (mock), 1 nM DHT, and/or 5 μM HF for 24 hours were subjected to RNA extraction and subsequent real-time RT-PCR. Expression of *ELK1* gene was normalized to that of *GAPDH*. Transcription amount is presented relative to that of mock treatment in each cell line. Each value represents the mean (+SD) from at least three independent experiments. *P < 0.05 (*vs*. mock treatment). **P < 0.01 (*vs*. mock treatment). ***P < 0.001 (*vs*. mock treatment).

We assessed the effects of androgen on ELK1 expression by reverse transcription (RT)-polymerase chain reaction (PCR), western blotting, and immunofluorescence in bladder cancer cells treated with DHT and/or an AR antagonist hydroxyflutamide (HF). DHT increased *ELK1* gene expression in two AR-positive bladder cancer sublines, UMUC3-control-shRNA (3.4-fold) and 647V-AR (3.2-fold), but not in AR-negative sublines (Figure 3A). Similarly, induction of ELK1 protein expression by DHT treatment was seen in UMUC3 and 647V-AR cells (Figure 3B). As expected, HF showing marginal agonist activity could abolish the effects of DHT on ELK1 mRNA/protein expression. Subcellular localization of ELK1 was then examined in UMUC3 and 647V-AR by western blotting: treatment with DHT resulted in increases in nuclear ELK1 expression as well as decreases in cytoplasmic ELK1 expression, and HF antagonized the effects of DHT (Figure 3C). Promotion of nuclear translocation of ELK1 by androgen was further confirmed by immunofluorescence (Figure 3D). ELK1-mediated transcriptional activity was also determined in the cell extracts with transfection of an ELK1 luciferase reporter plasmid and subsequent treatment with DHT and/or HF. DHT considerably augmented ELK1 luciferase activity, compared with mock treatment, and HF restored the enhancement (Figure 3E). To confirm the up-regulation of ELK1 activity by androgen, we measured the expression levels of c-fos, a downstream target of ELK1 signals [12]. Significant increases in *c-fos* gene expression by DHT were also seen in UMUC3-control-shRNA and 647V-AR, but not in UMUC3-AR-shRNA and 647V-control, which was inhibited by the addition of HF (Figure 3F). These results indicate that androgen-mediated AR signals up-regulate the expression and activity of ELK1 in bladder cancer cells.

### Role of ELK1 in bladder cancer cell proliferation

**Figure 4:**
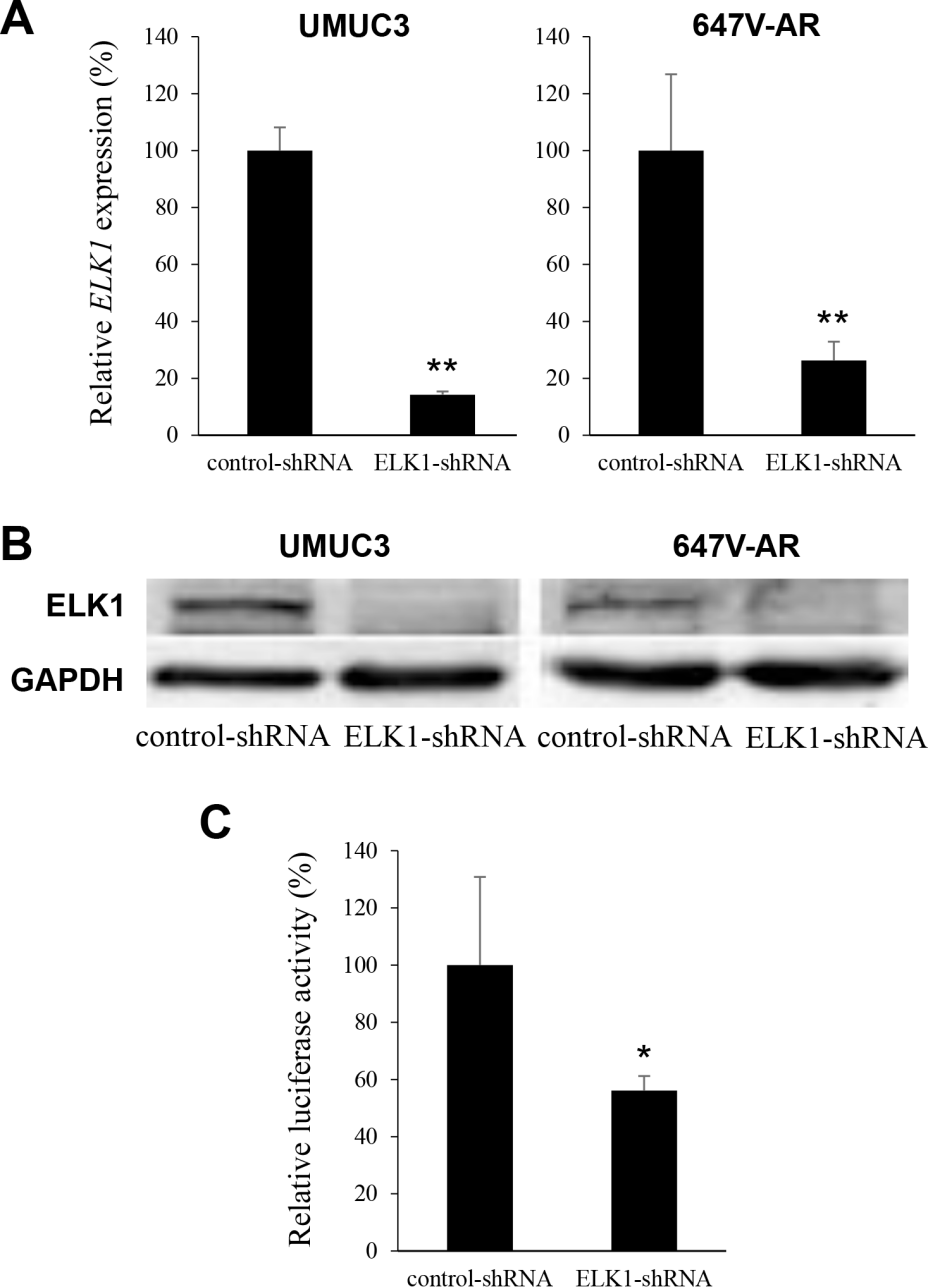
Silencing of ELK1 in bladder cancer cells **A.** Quantitative RT-PCR of *ELK1* in UMUC3-control-shRNA/ELK1-shRNA and 647V-AR-control-shRNA/ELK1-shRNA. Expression of *ELK1* gene was normalized to that of GAPDH. Transcription amount is presented relative to that of control-shRNA expression in each cell line. Each value represents the mean (+SD) from at least three independent experiments. **B.** Western blotting of ELK1 in UMUC3-control-shRNA/ELK1-shRNA and 647V-AR-control-shRNA/ELK1-shRNA. Cell extracts were immunoblotted for ELK1 (62 kDa). GAPDH (37 kDa) served as an internal control. **C.** ELK1 luciferase reporter activity in UMUC3 transfected with pELK1-Luc, pRL-TK, and control- or ELK1-shRNA. Luciferase activity is presented relative to that of control-shRNA expression. Each value represents the mean (+SD) from at least three independent experiments. *P < 0.05 (*vs*. control-shRNA). **P < 0.01 (*vs*. control-shRNA).

To further study the functional role of ELK1 in the growth of bladder cancer, an ELK1-shRNA was stably and transiently expressed in UMUC3 and 647V-AR cells, respectively. As expected, the levels of ELK1 mRNA (Figure 4A) and protein (Figure 4B) were substantially lower in ELK1-shRNA-expressing lines than in respective scrambled control-shRNA-expressing lines. ELK1 transcriptional activity was also diminished in UMUC3-ELK1-shRNA, compared with UMUC3-control-shRNA (Figure 4C).

**Figure 5:**
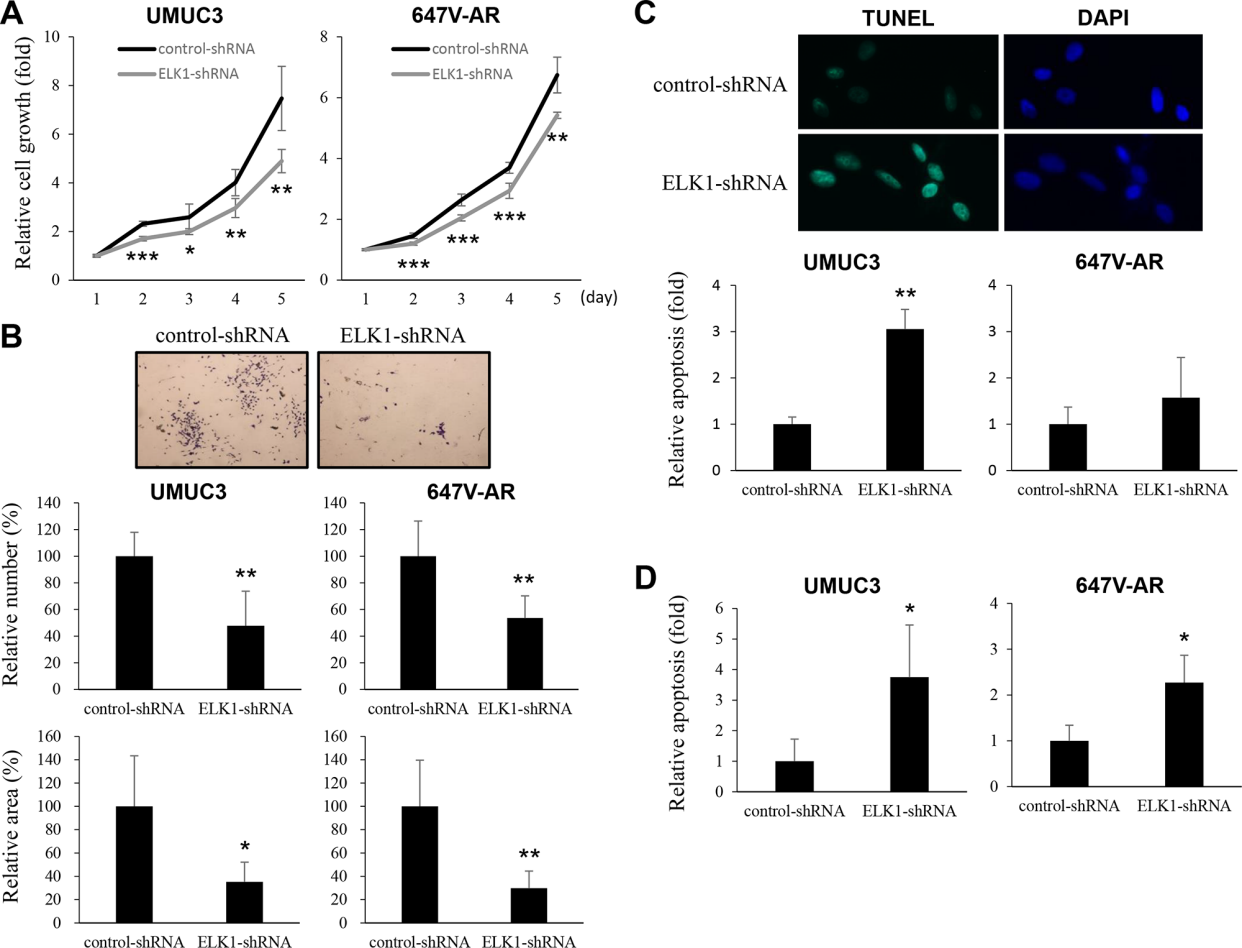
Effects of ELK1 inactivation on bladder cancer cell proliferation **A.** MTT assay in UMUC3-control-shRNA/ELK1-shRNA and 647V-AR-control-shRNA/ELK1-shRNA cultured for 1–5 days. Cell viability is presented relative to that of each control line at day 1. Each value represents the mean (+SD) from at least three independent experiments. **B.** Clonogenic assay in UMUC3-control-shRNA/ELK1-shRNA cultured for 2 weeks. The number of colonies and their areas quantitated, using the ImageJ software, are presented relative to those of each control line. Each value represents the mean (+SD) from at least three independent experiments. TUNEL assay **C.** and flow cytometry **D.** in UMUC3-control-shRNA/ELK1-shRNA and 647V-AR-control-shRNA/ELK1-shRNA. Apoptosis is presented relative to that of each control line. Each value represents the mean (+SD) from at least three independent experiments. *P < 0.05 (*vs*. control-shRNA). **P < 0.01 (*vs*. control-shRNA). ***P < 0.001 (*vs*. control-shRNA).

To determine whether ELK1 down-regulation exerts an influence on the proliferation of bladder cancer cells, we compared cell viability [by methyl thiazolyl disphenyl tetrazolium bromide (MTT) assay] and colony formation (by clonogenic assay) between ELK1-positive lines versus their knockdown lines. In UMUC3 (34% decrease at day 5) and 647V-AR (20% decrease at day 5) cells, the expression of ELK1-shRNA strongly suppressed their growth at days 2–5 (Figure 5A). Similarly, ELK1 silencing resulted in significant decreases in the number and area of colonies in these cells (Figure 5B).

To investigate how ELK1 regulates cell proliferation, we performed terminal deoxynucleotidyl transferase-mediated dUTP nick end labeling (TUNEL) assay (Figure 5C) and flow cytometry (Figure 5D) in ELK1-shRNA-expressing lines versus control-shRNA-expressing lines. In both assays, ELK1 knockdown was found to significantly induce apoptosis. However, it only marginally changed the cell cycle (*e.g*. G0/G1 population) (data not shown).

### Role of ELK1 on bladder cancer cell migration and invasion

**Figure 6:**
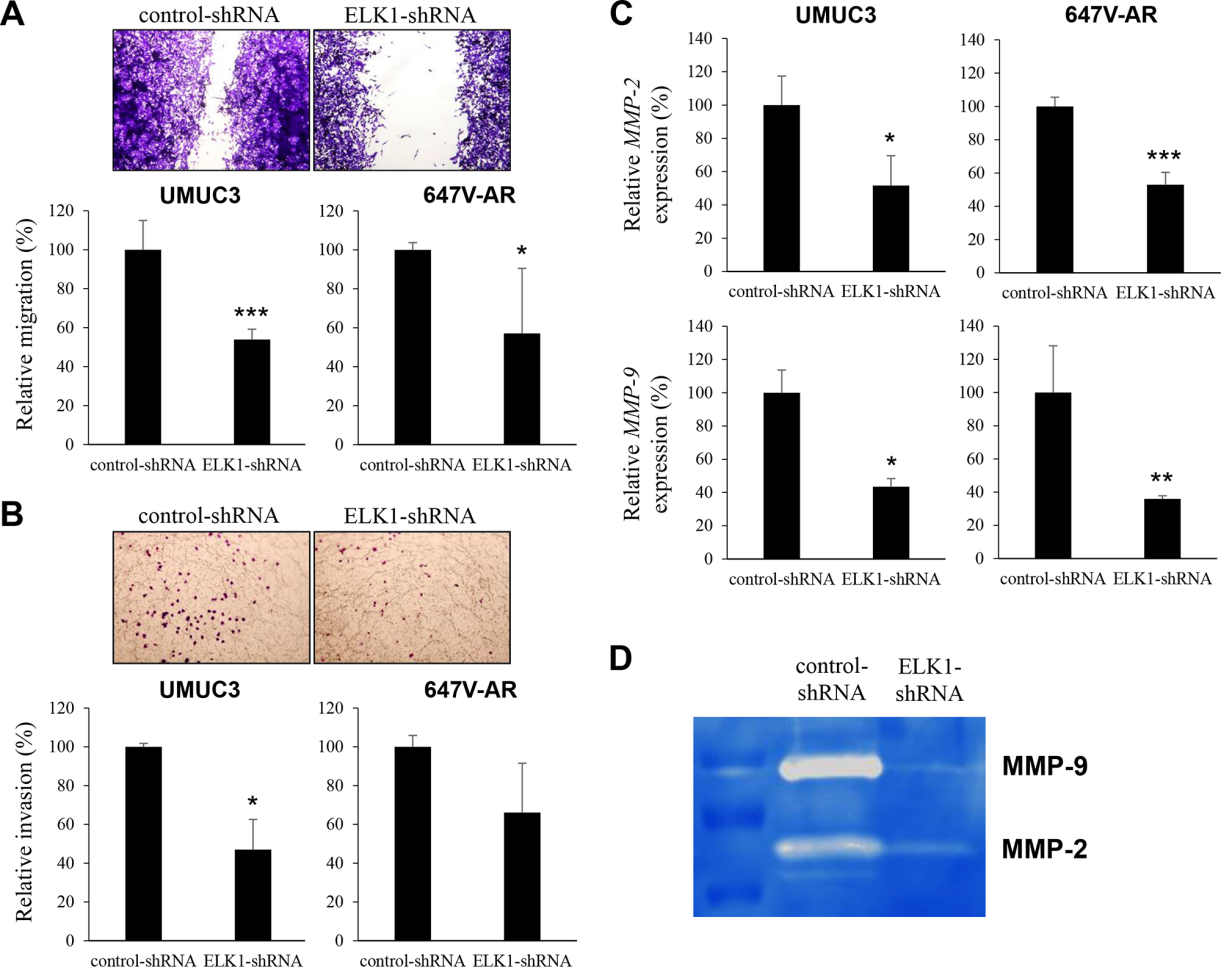
Effects of ELK1 inactivation on bladder cancer cell migration and invasion **A.** Wound healing assay in UMUC3-control-shRNA/ELK1-shRNA and 647V-AR-control-shRNA/ELK1-shRNA. The cells grown to confluence were gently scratched and the wound area was measured after 24-hour culture. The migration determined by the rate of cells filling the wound area is presented relative to that of each control line. Each value represents the mean (+SD) from at least three independent experiments. **B.** Transwell invasion assay in UMUC3-control-shRNA/ELK1-shRNA and 647V-AR-control-shRNA/ELK1-shRNA cultured in the Matrigel-coated transwell chamber. The number of invaded cells present in the lower chamber was counted under a light microscope (100x objective in five random fields). Cell invasion is presented relative to that of each control line. Each value represents the mean (+SD) from three independent experiments. **C.** Quantitative RT-PCR of *MMP-2* and *MMP-9* in UMUC3-control-shRNA/ELK1-shRNA and 647V-AR-control-shRNA/ELK1-shRNA. Expression of each specific gene was normalized to that of *GAPDH*. Transcription amount is presented relative to that of each control line. Each value represents the mean (+SD) from at least three independent experiments. **D.** Gelatin zymography in UMUC3-control-shRNA/ELK1-shRNA. The activity of MMP-2 or MMP-9 was indicated by clear zones of gelatin lysis against a blue background of stained substrate. *P < 0.05 (*vs*. control-shRNA). **P < 0.01 (*vs*. control-shRNA). ***P < 0.001 (*vs*. control-shRNA).

Cell migration and invasion are critical steps during tumor progression and metastasis. To see if ELK1 is involved in bladder cancer cell migration and invasion, we performed a scratch wound healing assay and a transwell invasion assay, respectively, in UMUC3 and 647V-AR expressing either ELK1-shRNA or control-shRNA. In the wound healing assay, silencing of ELK1, compared with control cells, significantly delayed wound closure 24 hours after wound generation (Figure 6A). Similarly, in the transwell assay, knockdown of ELK1 demonstrated marked decreases in cell invasion ability, compared with control lines (Figure 6B). Less significant reduction in 647V-AR cell migration/invasion, as well as cell proliferation, by the ELK1-shRNA might be due to its transient transfection (*vs*. stable expression in UMUC3 cells).

Using a quantitative RT-PCR method, we then assessed the effects of ELK1 silencing on the expression of MMPs that are known to play a critical role in cancer cell migration/invasion, angiogenesis, and resultant tumor progression and metastasis. ELK1-shRNA reduced the levels of *MMP-2* and *MMP-9* expression, compared with control-shRNA, in two cell lines (Figure 6C). We also determined the enzymatic activity of MMP-2 and MMP-9 by gelatin zymography that showed considerable decreases in their activity in UMUC3-ELK1-shRNA cells, compared with UMUC3-control-shRNA cells (Figure 6D).

### Requirement of activated AR to regulate bladder cancer cell growth by ELK1

**Figure 7:**
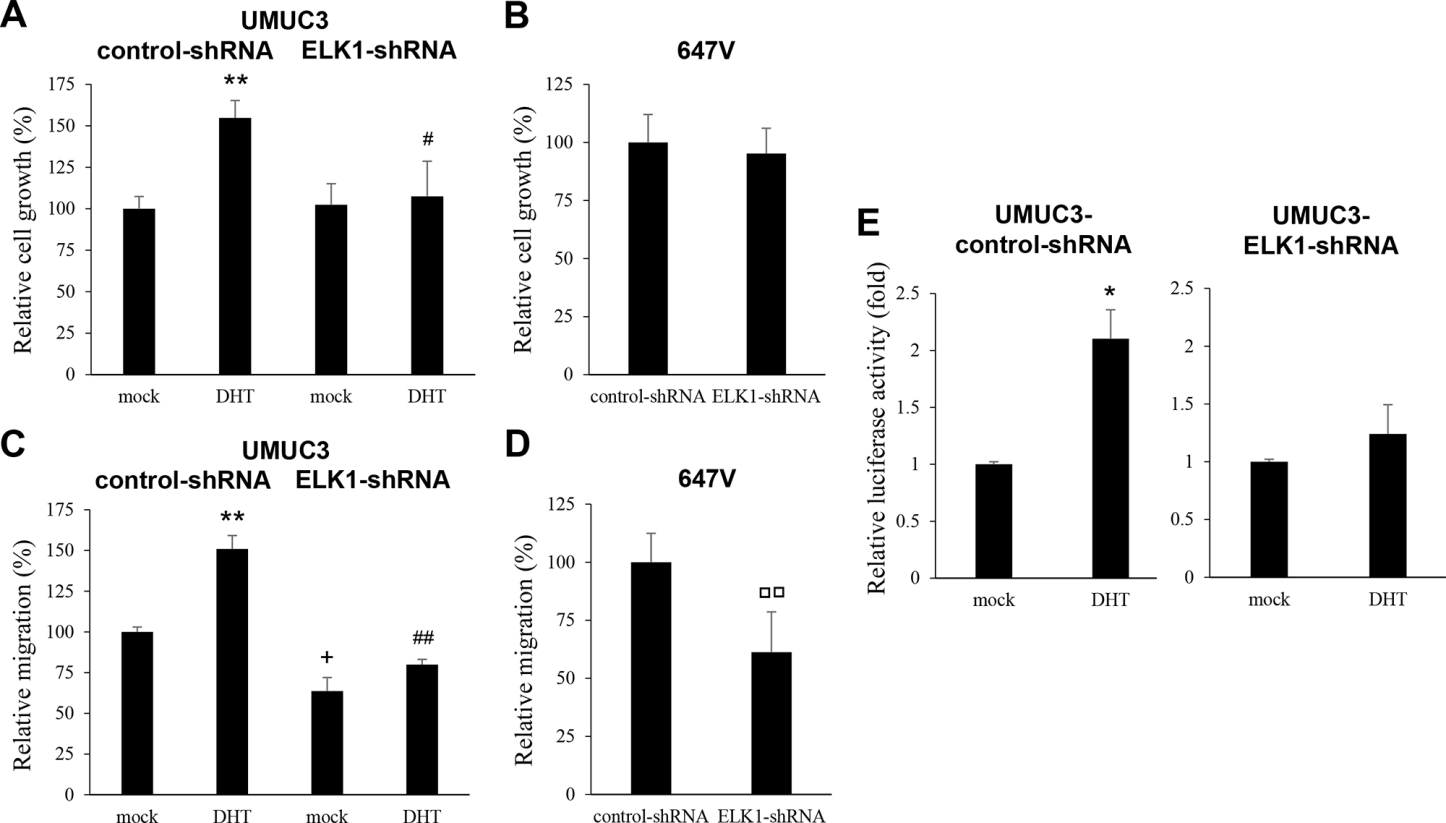
Effects of androgen on the proliferation or AR activity in ELK1 knockdown bladder cancer cells MTT assay in UMUC3-control-shRNA/ELK1-shRNA cultured for 5 days in the presence of ethanol (mock) or 1 nM DHT **A.** or in 647V-control-shRNA/ELK1-shRNA cultured for 5 days **B.** Cell viability is presented relative to that of mock-treated control line (A, lane 1) or that of control line (B) Each value represents the mean (+SD) from at least three independent experiments. Wound healing assay in UMUC3-control-shRNA/ELK1-shRNA in the presence of ethanol (mock) or 1 nM DHT **C.** in 647V-control-shRNA/ELK1-shRNA **D.** The cells grown to confluence were gently scratched and the wound area was measured after 24-hour culture. The migration determined by the rate of cells filling the wound area is presented relative to that of mock-treated control line (C, lane 1) or that of control line (D) Each value represents the mean (+SD) from at least three independent experiments. **E.** ELK1 luciferase reporter activity in UMUC3-control-shRNA and UMUC3-ELK1-shRNA transfected with pELK1-Luc and pRL-TK and subsequently cultured in the presence of ethanol (mock) or 1 nM DHT. Luciferase activity is presented relative to that of each line with mock treatment. Each value represents the mean (+SD) from at least three independent experiments. *P < 0.05 (mock *vs*. DHT in each line). **P < 0.01 (mock *vs*. DHT in each line). ^#^*P* < 0.05 (control- *vs*. ELK1-shRNA lines with DHT). ^##^*P* < 0.01 (control- *vs*. ELK1-shRNA lines with DHT). ^+^*P* < 0.05 (control- *vs*. ELK1-shRNA lines with mock treatment). ^□□^*P* < 0.01 (control- *vs*. ELK1-shRNA lines).

It has been demonstrated that ELK1-shRNA expression in LNCaP prostate cancer cells does not decrease, rather does increase modestly, their proliferation in an androgen depleted condition and that androgen does not significantly induce the proliferation of LNCaP-ELK1-shRNA cells [19]. We therefore investigated whether ELK1 could affect the viability and migration of AR-positive bladder cancer cells in the absence of androgens and whether androgen could enhance the viability and migration of AR-positive ELK1-shRNA-expressing bladder cancer cells. Consistent with our previous observations [2, 20], 1 nM DHT significantly increased control UMUC3 cell growth by 53% (Figure 7A; lanes 1 *vs*. 2). In contrast, DHT only marginally increased UMUC3-ELK1-shRNA cell growth by 5% (lanes 3 *vs*. 4). Additionally, similar to the findings in LNCaP [19] or UMUC3 cultured with normal fetal bovine serum (FBS) (Figure 5A), ELK1 silencing in UMUC3 cells cultured with charcoal-stripped FBS and 1 nM DHT significantly reduced their viability by 30% (lanes 2 *vs*. 4), whereas, in UMUC3 cells with charcoal-stripped FBS and no additional androgens, ELK1-shRNA only marginally affected their growth (2% increase; lanes 1 *vs*. 3). In AR-negative 647V cells, ELK1-shRNA also marginally (5%) inhibited their growth (Figure 7B). In a scratch would healing assay (Figure 7C), DHT accelerated wound closure of UMUC3-control-shRNA significantly (51% increase; *P* = 0.004; lanes 1 *vs*. 2) and that of UMUC3-ELK1-shRNA less significantly (25% increase; *P* = 0.063; lanes 3 *vs*. 4). Moreover, ELK1 silencing in UMUC3 cells cultured with charcoal-stripped FBS without and with 1 nM DHT retarded wound closure by 36% (*P* = 0.010; lanes 1 *vs*. 3) and 47% (*P* = 0.002; lanes 2 *vs*. 4), respectively. In 647V cells, ELK1-shRNA still inhibited their migration by 39% (*P* = 0.005; Figure 7D). Androgen-mediated AR transcriptional activities were also compared between control and ELK1 knockdown cell lines. DHT was found to induce AR transactivation by 2.10-fold in control UMUC3 cells but by only 1.24-fold in UMUC3-ELK1-shRNA cells (Figure 7E).

### Anti-tumor activity of ELK1 silencing *in vivo*

**Figure 8:**
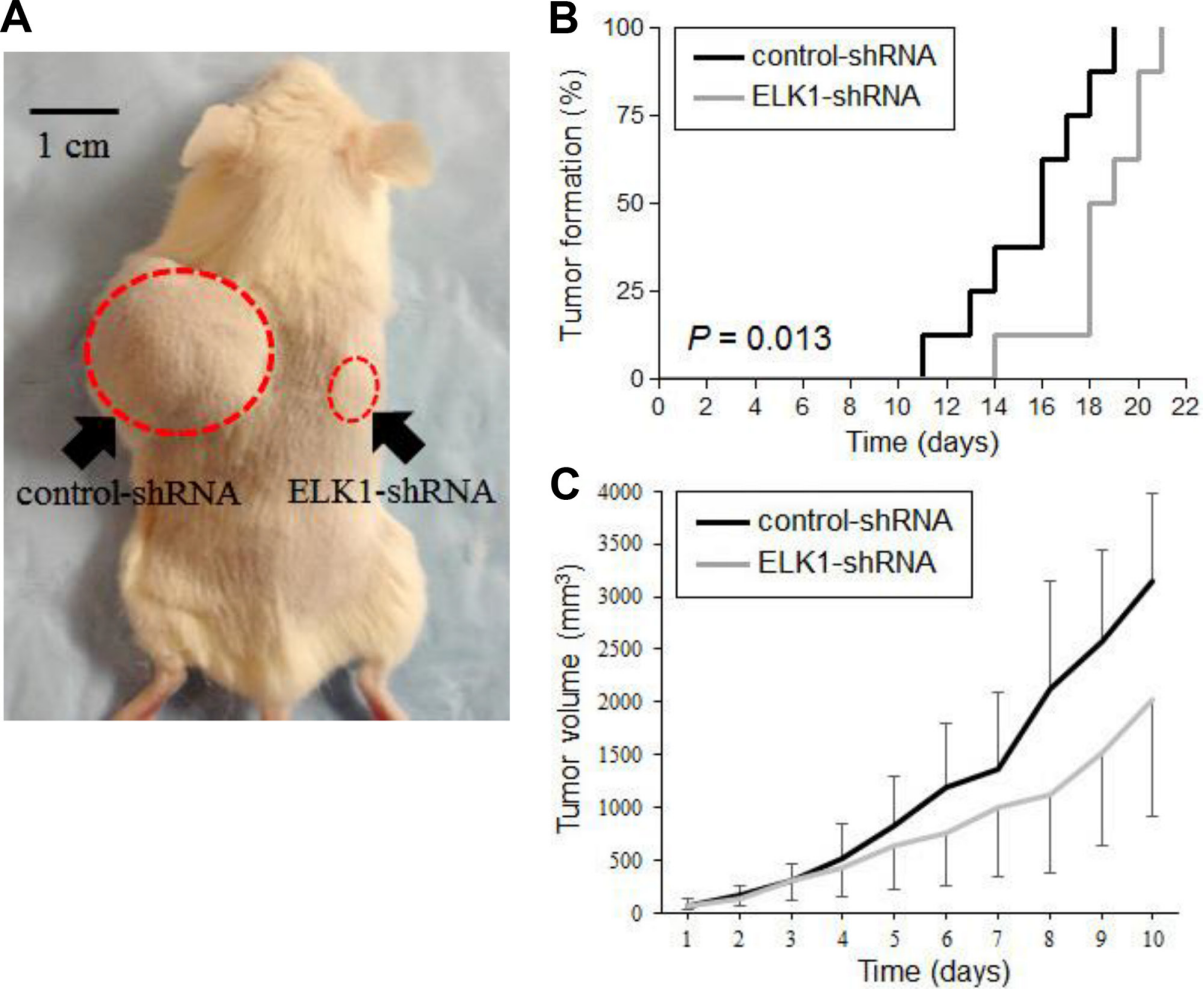
Effects of ELK1 inactivation on tumor growth in mouse xenograft models for bladder cancer **A.** UMUC3-control-shRNA/ELK1-shRNA cells were implanted subcutaneously into the left/right flanks of NOD-SCID mice, respectively, and tumor formation and its growth were monitored. **B.** Kaplan-Meier curves and log-rank test according to the endpoint set as tumor volume exceeding 40 mm^3^. **C.** Tumor size (estimated volume of each tumor exceeded 40 mm^3^ at day 0) was subsequently monitored every day. Each value represents the mean (+SD or –SD).

Finally, we used mouse xenograft models to investigate the role of ELK1 in bladder tumor outgrowth *in vivo*. UMUC3-control-shRNA and UMUC3-ELK1-shRNA cells were implanted subcutaneously into the flanks of immunocompromised mice (Figure 8A), and tumor development was monitored at the outset. ELK1 knockdown strikingly delayed the formation of xenograft tumors, compared with the control (Figure 8B). Following tumor formation (*i.e*. day 0 when the estimated tumor volume reached 40 mm^3^), its size was further monitored. As shown in Figure 8C, the inoculated ELK1-shRNA tumors were smaller than control-shRNA tumors, especially after day 5, although the differences in tumor size between the two groups did not narrowly reach statistical significance (*P* > 0.1 at days 1–8; *P* = 0.056 at day 9; *P* = 0.069 at day 10). These *in vivo* data further suggest that ELK1 silencing inhibits the development and progression of bladder cancer.

## DISCUSSION

Androgen-induced AR activation has been demonstrated to correlate with the promotion of bladder cancer progression [2–9]. Nonetheless, classical androgen-regulated genes, such as prostate-specific antigen and NKX3-1, which are known to involve the outgrowth of prostate cancer, do not significantly contribute to that of bladder cancer [21, 22]. In addition, molecular mechanisms of how androgens activate the AR pathway in bladder cancer cells are to be further investigated. To identify downstream targets of AR signals regulated by androgens in bladder cancer cells, we first screened 96 known transcription factors, using a profiling array kit. In UMUC3 cells with versus without R1881 treatment, the expression of at least six transcription factors was found to be significantly up-regulated. However, the stimulatory effect of DHT was not significant in UMUC3-control-shRNA cells (*e.g*. NFAT, NKX2–5, GLI1, MyoD) or remained significant in UMUC3-AR-shRNA cells (*e.g*. ROR). We thus narrowed down the candidate to ELK1 and further investigated whether ELK1 could be activated by androgen-mediated AR signaling in bladder cancer cells and thereby affected tumor progression. Meanwhile, up-regulation of ROR in AR knockdown cells may imply androgen action via the non-AR pathway or the presence of the second AR in bladder cancer cells, which requires further studies.

In accordance with the above findings, DHT activated ELK1 in AR-positive bladder cancer cells. However, some of our findings in bladder cancer were inconsistent with those shown in prostate cancer [19]. For instance, androgens induced the expression of ELK1 and its downstream target c-fos in bladder cancer cells, while they failed to alter their expression significantly in prostate cancer cells. Instead, in prostate cancer cells, AR was shown to function as a co-activator of ELK1 via bypassing the classical mechanism of ELK1 activation by phosphorylation. In AR-positive osteoblast cells, DHT was shown to increase the phosphorylation of ELK1 [23]. Androgens could also activate a variety of target genes in ELK1-dependent manners and enhanced ELK1 promoter activity in prostate cancer cells [19]. We additionally found in bladder cancer cells that androgens induced nuclear translocation of ELK1 and its transactivation.

ELK1 is involved in a wide variety of functions, including the regulation of cell proliferation, cell cycle control, and apoptosis [24–26]. AR is also known to induce MAPK signaling cascades, leading to cell proliferation [27–29]. In prostate cancer, ELK1-shRNA expression resulted in a significant decrease in the growth of androgen-sensitive/AR-positive LNCaP cells cultured with R1881, but not in that of LNCaP cultured without androgens or that of AR-negative cells [19]. Consistent with the findings in prostate cancer cells, we observed strong inhibition in the proliferation and colony formation of AR-positive bladder cancer cells in the presence of androgen (*i.e.* normal FBS, DHT treatment), but not in the former of AR-positive cells in androgen-depleted conditions or that of AR-negative cells, by ELK1 knockdown. Thus, AR activation is likely required for the regulation of bladder cancer cell growth by ELK1. We also demonstrated that androgens failed to significantly increase the viability of bladder cancer cells expressing ELK1-shRNA, suggesting ELK1-dependent effects of androgens on cell proliferation. We additionally found that ELK1 silencing induced apoptosis of bladder cancer cells that possessed a functional AR and were cultured with FBS containing androgens but did not modulate the cell-cycle status. In contrast, in breast cancer MCF-7 cells expressing an AR as well as estrogen receptors, ELK1 overexpression reduced their colony formation [25]. Indeed, ELK1-shRNA modestly increased the cell growth of AR-positive prostate cancer [19] as well as bladder cancer (Figure 7A) in androgen-depleted conditions.

As aforementioned, ELK1 has been implicated in cell migration and invasion via modulating the expression of various genes/proteins important in them, including MMP-2 and MMP-9. The role of ELK1 in promoting the migration of mammary gland-derived cells [15, 17] as well as gastric cancer cells [16] has indeed been confirmed. In these studies [15–17], however, the status of AR expression in the cell lines used and the effect of androgens on ELK1-mediated cell migration were not investigated. Instead, in cardiac fibroblast cells, estrogen was shown to modulate MMP-2 promoter activity via phosphorylation of ELK1 [30]. In the current study, ELK1 silencing in AR-positive bladder cancer lines cultured with androgen resulted in significant decreases in cell migration and invasion as well as the expression and enzymatic activity of MMP-2/MMP-9. Importantly, the inhibitory effects of ELK1-shRNA expression on cell migration were also seen in an AR-positive line cultured in an androgen-depleted condition as well as in an AR-negative line. Thus, in contrast to its function in cell proliferation, ELK1 may not require activated AR to regulate bladder cancer cell migration. We observed similar findings in prostate cancer lines: ELK1-shRNA strongly inhibited AR-negative cell migration/invasion and MMP-2/MMP-9 expression (unpublished data). Nonetheless, DHT more significantly induced cell migration of UMUC3-control-shRNA, compared with UMUC3-ELK1-shRNA, suggesting that the effect of androgens could be mediated at least partially through the ELK1 pathway.

Again, the interaction between AR and ELK1 signals has been studied in prostate cancer cells [19]. Our data in bladder cancer cells indicate that androgen induces the expression and activity of ELK1. Of note, activated AR is necessary for ELK1 to promote bladder cancer cell proliferation, but not cell migration. Similarly, androgen appears to require ELK1 to induce bladder cancer cell proliferation, while it can still increase the migration ability of ELK1-knockdown cells. In addition, androgen does not significantly induce AR transcriptional activity in the absence of ELK1, suggesting its necessity for AR activation. Thus, ELK1 and AR signals appear to require each other for their functions at least in bladder cancer cell proliferation.

The expression of ETS-domain proteins has been demonstrated in several types of malignancies, including colon cancer, Ewing sarcoma, leukemias, breast cancer, and cervical carcinoma [31]. Elevated expression of ELK1 or p-ELK1 has also been detected in tissue specimens of breast cancer [32, 33], colonic adenocarcinoma [34], and lung non-small cell carcinoma [35, 36]. Particularly in breast cancer tissues, positive correlations between p-ELK1 expression and AR [32] or estrogen receptor [33] expression were noted. However, none of these studies have assessed the prognostic value of ELK1 or p-ELK1 expression. On the other hand, the expression levels of ELK1 [37] and p-ELK1 [38] have been determined in bladder cancer cell lines. Our immunohistochemical staining in bladder tissue microarrays (TMAs) showed that, compared with non-neoplastic urothelium, significant increases in the expression of ELK1 and p-ELK1 were observed in bladder tumor. Furthermore, although the levels of ELK1 and p-ELK1 expression were not correlated with tumor grades or stages, p-ELK1 positivity precisely predicted recurrence of non-muscle invasive tumor in a univariate setting as well as a poor prognosis of muscle-invasive tumor after radical cystectomy both in univariate and multivariate settings. Thus, p-ELK1 may serve as a prognosticator especially in patients with muscle-invasive bladder cancer. These results also suggest that ELK1 activation promotes bladder cancer growth. More interestingly, ELK1/p-ELK1 and AR expression in bladder tumors was positively correlated.

In conclusion, ELK1 likely plays an important role in bladder tumorigenesis and cancer progression. ELK1 is indeed activated in bladder cancer, which is further induced by AR activation. It is thus possible that ELK1 and AR serve each other as transcriptional coactivators in bladder cancer cells. Accordingly, not only AR or ELK1 signaling but also their interaction offers a therapeutic target for bladder cancer.

## MATERIALS AND METHODS

### Cell culture and chemicals

SVHUC, UMUC3, TCCSUP, and 5637 cell lines were originally obtained from the American Type Culture Collection. 647V cell line was used in our previous studies [2, 39, 40]. All these lines were recently authenticated, using GenePrint 10 System (Promega), by the institutional core facility. SVHUC cells and other cell lines were maintained in F-12K (Mediatech) and Dulbecco's modified Eagle's medium (Mediatech), respectively, all supplemented with 10% FBS, penicillin (100 units/mL), and streptomycin (100 units/mL) at 37°C in a humidified atmosphere of 5% CO_2_. Cells were cultured in phenol red-free medium supplemented with either 5% FBS or 5% charcoal-stripped FBS (for androgen treatment) at least 24 hours before experimental treatment. We obtained R1881 from PerkinElmer; and DHT and HF from Sigma.

### Stable cell lines

A shRNA plasmid targeting human ELK1 (sc-35290-SH; Santa Cruz Biotechnology) or a non-silencing control shRNA plasmid (sc-108060; Santa Cruz Biotechnology) was transfected, using Lipofectamine^®^ 2000 transfection reagent (Life Technologies). Selection of stable clones was carried out with puromycin (Sigma) treatment at a concentration of 6 μg/mL. UMUC3-AR-shRNA and UMUC3-control-shRNA, using a retrovirus vector pMSCV/U6, as well as 647V-AR expressing a full-length wild-type human AR and its control line (647V-vector), using a lentivirus vector (pWPI-AR or pWPI-control) with psPAX2 envelope and pMD2.G packaging plasmids, were established in our previous studies [5, 39].

### Transcription factor profiling assay

Nuclear extracts isolated from UMUC3 cells treated with or without 1 nM R1881 for 24 hours were analyzed, using an assay kit (TF Activation Profiling Plate Array II, Signosis), according to the manufacturer's instructions. Chemiluminescence was measured by a multidetection microplate reader.

### Bladder TMA and IHC

We retrieved bladder tissue specimens obtained by transurethral resection performed at the Johns Hopkins Hospital. All the sections were reviewed for confirmation of original diagnoses, according to the 2004 World Health Organization/International Society of Urological Pathology classification system for urothelial neoplasms. Appropriate approval from the institutional review board was obtained before construction and use of the TMA. Bladder TMAs, consisting of 129 cases of urothelial neoplasm, were constructed from formalin fixed paraffin embedded specimens, as described previously [41, 42]. These patients included 98 men and 31 women with a mean/median age of 65.7/69 years (range: 26–89). The primary tumors included 11 papillary urothelial neoplasms of low malignant potential (PUNLMPs), 40 non-invasive (pTa) low-grade urothelial carcinomas, 27 non-muscle-invasive (pTa or pT1) high-grade urothelial carcinomas, and 51 muscle-invasive (≥pT2) high-grade urothelial carcinomas. All 51 patients with muscle-invasive tumor ultimately underwent cystectomy. None of the patients had received therapy with radiation or anti-cancer drugs prior to the collection of the tissues included in the TMAs. All of these 129 cases were included in our prior study analyzing 188 cases for the expression of AR [41].

IHC was performed on the sections (5 μm thick) from the bladder TMAs, as described previously [5, 42]. Briefly, after deparaffinization, hydration, and antigen retrieval, samples were incubated overnight at 4°C with a primary antibody to ELK1 (clone I-20; dilution 1:50; Santa Cruz Biotechnology) or p-ELK1 (clone B-4 phosphorylated at serine 383; dilution 1:30; Santa Cruz Biotechnology) and then with a broad spectrum secondary antibody (Invitrogen). All stains were manually quantified by a single pathologist (H.M.) blinded to sample identify. The German immunoreactive scores calculated by multiplying the percentage of immunoreactive cells (0% = 0; 1–10% = 1; 11–50% = 2; 51–80% = 3; 81–100% = 4) by staining intensity (negativ*e* = 0; wea*k* = 1; moderat*e* = 2; stron*g* = 3) were considered negative (0; 0–1), weakly positive (1+; 2–4), moderately positive (2+; 6–8), and strongly positive (3+; 9–12).

### RT and real-time PCR

Total RNA (0.5 μg) isolated from cultured cells, using TRIzol (Invitrogen), was reverse transcribed using 1 μM oligo (dT) primers and 4 units of Omniscript reverse transcriptase (Qiagen) in a total volume of 20 μL. Real-time PCR was then performed, using SYBR GreenER qPCR superMix (Bio-Rad) for iCycler (Bio-Rad), as described previously [5, 8, 39]. The following primer pairs were used for RT-PCR: *ELK1* (forward, 5′-CAGCCAGAGGTGTCTGTTACC-3′; reverse, 5′-GAG CGCATGTACTCGTTCC-3′), *c-fos* (forward 5′-CGAG ATGGAGATCGGTATGGT-3′; reverse, 5′-GGGTCTTC TTACCCGGCTTG-3′); *MMP-2* (forward, 5′-TACAGGA TCATTGGCTACACACC-3′; reverse, 5′-GGTCACATC GCTCCAGACT-3′); and *MMP-9* (forward, 5′-TGT ACCGCTATGGTTACACTCG-3′; reverse, 5′-GGCA GGGACAGTTGCTTCT-3′). *GAPDH* (forward, 5′-CTCCT CCACCTTTGACGCTG-3′; reverse, 5′-CATACCAGG AAATGAGCTTGACAA-3′) was used as an internal control.

### Western blotting

Protein extraction and western blotting were performed, as described previously [40] with minor modifications. We also used a nuclear and cytoplasmic extraction reagent kit (NE-PER, Thermo Scientific) for obtaining separate nuclear and cytoplasmic fractions. Equal amounts of protein (30–50 μg) obtained from cell extracts were separated in 10% sodium dodecyl sulfate (SDS)-polyacrylamide gel electrophoresis (PAGE) and transferred to polyvinylidene difluoride membrane (Immun-Blot PVDF Membrane, Bio-Rad) by electroblotting. Specific antibody binding was detected, using an anti-ELK1 antibody (clone I-20; dilution 1:50), an anti-GAPDH antibody (clone 6C5; dilution 1:5000; Santa Cruz Biotechnology), or an anti-histone H1 antibody (clone FL-219; dilution 1:1000; Santa Cruz Biotechnology), and a secondary antibody (mouse IRDye 680LT or rabbit IRDye 800CW, LI-COR,), followed by scanning with an infrared imaging system (Odyssey, LI-COR).

### Immunofluorescent staining

Cells plated onto 8-well chamber slides (NuncLab-Tek, Thermo Scientific) were cultured in medium containing ethanol, DHT, and/or HF for 24 hours. At the end of the drug treatment, the adherent cells were rinsed and fixed by 4% paraformaldehyde. The cells were then blocked with 1% bovine serum albumin for 1 hour at 37°C, and a primary antibody (ELK1; clone I-20; dilution 1:50) was added and incubated for 1 hour at 37°C. Fluorescence images were acquired with a fluorescence microscopy (EVOS FL Auto, Life Technologies).

### Reporter gene assay

Cells at a density of 50–70% confluence in 24-well plates were co-transfected with 250 ng of pELK-Luc reporter plasmid DNA (LR-2061, Signosis) and 2.5 ng of pRL-TK plasmid DNA, using GeneJuice (Novagen). After 18 hours of transfection, the cells were cultured in the presence or absence of DHT and/or HF for 24 hours. Cell lysates were then assayed for luciferase activity determined using a Dual-Luciferase Reporter Assay kit (Promega) and luminometer (FLUOstar Omega, BMG Labtech).

### Cell proliferation

We used MTT assay to assess cell viability, as described previously [40]. Briefly, cells (0.5–1 × 10^3^) seeded in 96-well tissue culture plates were incubated for up to 120 hours, and at the end of the culture 10 μL MTT stock solution (5 mg/mL; Sigma) was added to each well with 100 μL of medium for 4 hours at 37°C. The medium was replaced with 100 μL dimethyl sulfoxide, followed by incubation for 5 minutes at room temperature. The absorbance was then measured at a wavelength of 570 nm with background subtraction at 655 nm using luminometer (FLUOstar Omega).

### Colony formation

Cells (5 × 10^2^) seeded in 12-well plates were allowed to grow until colonies in the control well were easily distinguishable. The cells were then fixed with methanol and stained with 0.1% crystal violet. The number of colonies and their areas were quantitated using ImageJ software (National Institutes of Health).

### Apoptosis and cell cycle analysis

The TUNEL assay was performed on cell-burdening coverslips, using the DeadEnd Fluorometric TUNEL system (Promega), followed by counterstaining for DNA with 4′,6′-diamidino-2-phenylindole (DAPI). Apoptotic index was determined in the cells visualized by the fluorescence microscopy (EVOS FL Auto). For cell cycle analysis, flow cytometry was performed in cells (1 × 10^6^/10-cm dish) cultured for 24 hours, harvested with trypsin, fixed in 70% ethanol, and stained with propidium iodide (PI) buffer (50 μg/mL) for 30 minutes. Cellular PI content was measured on a Guava PCA-96 Base System^TM^ flow cytometer (EMD Millipore) equipped with a green laser at 532 nm wavelength. Data were analyzed, using the Guava Cell Cycle software (EMD Millipore).

### Cell migration

In order to evaluate the ability of cell migration, a scratch wound healing assay was performed. Cells at a density of 90–100% confluence in 12-well plates were scratched manually with a sterile 200 μl plastic pipette tip, cultured for 24 hours, fixed with methanol, and stained with 0.1% crystal violet. The width of the wound area was quantitated, using ImageJ.

### Cell invasion

Cell invasiveness was determined, using a Matrigel (60 μg; BD Biosciences)-coated transwell chamber (8.0 μm pore size polycarbonate filter with 6.5 mm diameter; Corning), as described previously [40]. Briefly, cells (5 × 10^4^) in 100 μL of serum-free medium were added to the upper chamber of the transwell, whereas 600 μL of medium containing 5% FBS was added to the lower chamber. After incubation for 16 hours at 37°C in a CO_2_ incubator, invaded cells were fixed, stained with 0.1% crystal violet, and counted under a light microscope.

### Gelatin zymography

The gelatinolytic activity of MMPs was determined by SDS-PAGE gelatin zymography, as described previously [43]. Briefly, the conditioned medium derived from culture of cells at a density of 60–70% confluence in 10-cm dish in a serum-free condition for 24 hours was concentrated using Amicon Ultra-4 centrifugal filter units (30 kDa, Millipore) according to the manufacturer's instructions, followed by 10% SDS-PAGE in a gel containing 0.1% gelatin (Sigma). After electrophoresis, the gel was rinsed with a renaturing buffer (Bio-Rad) for 60 minutes, incubated overnight in a developing buffer (Bio-Rad) at 37°C with shaking, and stained with 0.25% Coomassie Brilliant Blue R-250 (Bio-Rad).

### Mouse xenograft model

Animal protocols in accordance with the National Institutes of Health Guidelines for the Care and Use of Experimental Animals were approved at our institution. Cells (5 × 10^5^/100 μL/site) resuspended in Matrigel (BD Biosciences) were subcutaneously injected into the flank of 6-week-old male immunocompromised NOD-SCID mice, as described previously [40, 43]. Serial caliper measurements of perpendicular diameters were used to calculate tumor volume by the following formula: (short diameter)^2^ × (longest diameter) × 0.5.

### Statistical analysis

The Fisher exact test or the χ^2^ test was used to evaluate the associations between categorized variables. The numerical data were compared by Student's *t*-test. Correlations between variables were determined by the Spearman's correlation coefficient. Survival rates in patients were calculated by the Kaplan-Meier method and comparison was made by log-rank test. These included comparisons among patients with non-muscle-invasive tumor or those with muscle-invasive tumor. Tumor progression was defined as development of high-grade or invasive carcinoma (initial PUNLMP or low-grade carcinoma), muscle-invasive or metastatic tumor (initial non-muscle-invasive high-grade carcinoma), or local recurrence or metastatic tumor after radical cystectomy (initial muscle-invasive tumor). In addition, the COX proportional hazards model (stepwise regression) was used to assess the prognostic indicators, including tumor grade, pathologic pT stage, lymph node involvement at cystectomy, lymphovascular invasion, concomitant urothelial carcinoma *in situ*, AR expression, ELK1 expression, and p-ELK1 expression. *P* values less than 0.05 were considered to be statistically significant.
